# Validation of Oxford nanopore sequencing for improved New World *Leishmania* species identification via analysis of 70-kDA heat shock protein

**DOI:** 10.1186/s13071-023-06073-9

**Published:** 2023-12-18

**Authors:** Luz Helena Patiño, Nathalia Ballesteros, Marina Muñoz, Jesús Jaimes, Adriana C. Castillo-Castañeda, Roy Madigan, Alberto Paniz-Mondolfi, Juan David Ramírez

**Affiliations:** 1 Centro de Investigaciones en Microbiología y Biotecnología-UR (CIMBIUR), Facultad de Ciencias Naturales, Universidad del Rosario, Bogotá, Colombia; 2 Animal Hospital of Smithson Valley, 286 Singing Oaks, Ste 113, Spring Branch, TX 78070 USA; 3 Molecular Microbiology Laboratory, Department of Pathology, Molecular and Cell-Based Medicine, Icahn School of Medicine at Mount Sinai, New York, NY 10029 USA

**Keywords:** *Leishmania*, MinION sequencing, HSP70-Long, HSP70-Short, Cutaneous Leishmaniasis

## Abstract

**Background:**

Leishmaniasis is a parasitic disease caused by obligate intracellular protozoa of the genus *Leishmania*. This infection is characterized by a wide range of clinical manifestations, with symptoms greatly dependent on the causal parasitic species. Here we present the design and application of a new 70-kDa heat shock protein gene (*hsp70*)-based marker of 771 bp (HSP70-Long). We evaluated its sensitivity, specificity and diagnostic performance employing an amplicon-based MinION™ DNA sequencing assay to identify different *Leishmania* species in clinical samples from humans and reservoirs with cutaneous leishmaniasis (CL) and visceral leishmaniasis (VL). We also conducted a comparative analysis between our novel marker and a previously published HSP70 marker known as HSP70-Short, which spans 330 bp.

**Methods:**

A dataset of 27 samples from Colombia, Venezuela and the USA was assembled, of which 26 samples were collected from humans, dogs and cats affected by CL and one sample was collected from a dog with VL in the USA (but originally from Greece). DNA was extracted from each sample and underwent conventional PCR amplification utilizing two distinct HSP70 markers: HSP70-Short and HSP70-Long. The subsequent products were then sequenced using the MinION™ sequencing platform.

**Results:**

The results highlight the distinct characteristics of the newly devised HSP70-Long primer, showcasing the notable specificity of this primer, although its sensitivity is lower than that of the HSP70-Short marker. Notably, both markers demonstrated strong discriminatory capabilities, not only in distinguishing between different species within the *Leishmania* genus but also in identifying instances of coinfection.

**Conclusions:**

This study underscores the outstanding specificity and effectiveness of HSP70-based MinION™ sequencing, in successfully discriminating between diverse *Leishmania* species and identifying coinfection events within samples sourced from leishmaniasis cases.

**Graphical abstract:**

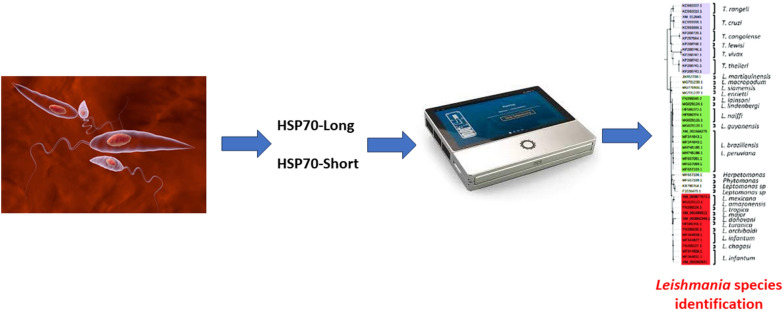

**Supplementary Information:**

The online version contains supplementary material available at 10.1186/s13071-023-06073-9.

## Background

Leishmaniases comprise a cluster of vector-borne ulcerative skin diseases triggered by parasites belonging to the genus *Leishmania*. The disease manifests in six distinct clinical forms, each characterized by the specific location of the parasite within the affected tissue. Among these forms, cutaneous leishmaniasis (CL) stands out as the most prevalent form of the infection, and visceral leishmaniasis (VL) has the distinction of being the most severe, pathogenic and clinically relevant manifestation across the disease spectrum [1]. To date, 53 distinct *Leishmania* species have been identified, of which 31 are considered to be as parasites affecting mammals, including humans. Among these latter 31 species, 20 are recognized as pathogenic to humans [2]. Several species of *Leishmania* are the causative agents of various clinical forms of the disease [3].

To date, various molecular techniques have been developed for the identification and differentiation of *Leishmania* species. These techniques rely on different DNA sequences as targets, including molecular elements such as minicircles kinetoplast DNA (kDNA) [4, 5], 18S ribosomal DNA (rDNA) [6], internal transcribed spacers (ITS1 and ITS2) [1, 7, 8], glucose-6-phosphate dehydrogenase (G6PD) [9], mannose phosphate isomerase genes (MPI) [8, 9], the miniexon (spliced leader) gene repeat [10], heat-shock proteins (HSPs) [1, 8] and cysteine proteinase B (CPB) [8]. The most widely used and prominent molecular methods currently available involve utilization of species-specific restriction sites via restriction fragment length polymorphism analysis (RFLPs) [11], the random amplified polymorphic DNA (RAPD) technique [12], high-resolution melting (HRM) [3, 13], quantitative PCR (qPCR) [5], multilocus sequence typing (MLST) [1], loop-mediated isothermal amplification (LAMP) [14] and Sanger sequencing. However, it is important to note that while these methods effectively differentiate *Leishmania* species, they can be costly, time-consuming and labor-intensive and require specialized laboratory techniques, equipment and robust infrastructure [15]. Moreover, some of these techniques might not be sensitive enough to detect low-level parasite DNA or identify coinfections involving multiple *Leishmania* species.

In recent years, next-generation sequencing (NGS), particularly in the form of amplicon-based sequencing, has emerged as a promising solution to overcome these limitations. Recent investigations have harnessed amplicon-based Illumina sequencing to detect and classify trypanosomatid species across various hosts, including mammals, human patients with CL and VL, as well as sand fly vectors. These studies have also delved into the identification of mixed trypanosomatid infections [16, 17].

Conversely, the capabilities of the Oxford Nanopore Technologies (Oxford, UK) MinION™ DNA sequencing system (referred to as MinION™ in following text) have been subjected to extensive scrutiny across numerous publications. These studies have illustrated the capacity of MinION™ to produce high-quality, single-contig microbial genomes [18], enhance eukaryotic genome assembly [19] and detect a wide array of pathogens, including SARS-CoV-2, Chikungunya virus, Hepatitis virus C, *Orthohantavirus* [20, 21], *Salmonella enterica* and *Salmonella typhimurium* [22, 23]. MinION™ has also proven instrumental in pinpointing drug resistance and pathogenicity biomarkers [24].

Nevertheless, the use of MinION™ specifically within the context of amplicon-based analysis, with a focus on identifying distinct *Leishmania* species from both cultured and biological samples, and the detection of coinfection events, remains relatively limited. In a recent study, a novel protocol was developed that employed a 234-bp marker of the heat-shock protein 70 gene (*hsp70*) to identify *Leishmania* species using MinION™ [15]. While this technique exhibited high sensitivity and proficiency in discriminating reference strains and clinical samples, concerns arose regarding whether the marker's length adequately covered the extent of nucleotide variations present among diverse *Leishmania* species.

The 70-kDa heat-shock proteins (HSP70) exhibit a high degree of conservation both in sequence and function across prokaryotes and eukaryotes. They hold significant importance as molecular chaperones, playing essential roles in protein folding and transport processes [25]. Over time, *hsp70* has emerged as the predominant target for identifying and distinguishing *Leishmania* species [26]. In addition, this gene has been instrumental in studies related to taxonomy and phylogeny [27–29], demonstrating remarkable congruence with multilocus enzyme electrophoresis (MLEE) typing, a widely acknowledged gold standard in *Leishmania* typing [30].

Given the widespread prevalence of leishmaniasis cases across the globe, coupled with the extensive array of vectors implicated in parasite transmission and the various species linked to distinct clinical presentations [31–33], it is imperative to develop rapid, precise and highly sensitive methods for detecting and identifying *Leishmania* species. Moreover, increases in the occurrences of coinfections among humans, reservoirs and vectors have been documented in recent times [16, 17, 34], accentuating the pressing need to optimize these efforts. The insights gleaned from such studies will not only bolster accurate diagnoses but also play a pivotal role in guiding therapeutic decisions. This, in turn, will aid in the selection of suitable antileishmanial agents and optimal treatment courses for patients.

In this context, we developed a 771-bp marker (HSP70-Long) that specifically targets *hsp70*. We meticulously evaluated its sensitivity, specificity and diagnostic efficacy by employing an amplicon-based MinION™ sequencing approach. This method was instrumental in identifying *Leishmania* species within clinical samples sourced from both humans and reservoirs afflicted by CL and VL. Additionally, we conducted a comprehensive performance comparison between our newly devised marker and a previously published HSP70 marker [35].

## Methods

### Design of HSP70-Long primers

To develop primers for the HSP70-Long, we established an in-house database leveraging prior research [16]. In brief, we systematically queried the NCBI Nucleotide database (https://www.ncbi.nlm.nih.gov/nuccore?term) to assemble all available HSP70 sequences pertaining to trypanosomatids. This targeted approach resulted in the retrieval of a comprehensive dataset comprising 1460 distinct sequences.

Subsequently, we refined our search outcomes by implementing specific filters. These filters encompassed molecule type categorization (genomic DNA/RNA), source database selection (GenBank) and sequence length delineation (ranging from 700 to 5000). We took care to exclude sequences that did not correspond to the *Leishmania* genus or lacked accurate taxonomic classification at the species level (designated as *Leishmania* spp.). Additionally, sequences exhibiting an excessive abundance of gaps within their content and those of low quality were omitted from consideration. The resultant pool of sequences that met these criteria was then subjected to alignment using the Clustal W algorithm, employing the UGENE version 33.0 software platform [36].

Our final reference database consisted of a total of 414 meticulously selected sequences. This comprehensive dataset can be readily accessed by the public through the following link: https://github.com/gimur/-Enhancing-Leishmania-Species-Identification-from-Clinical-Samples. Next, employing DnaSP software version 5.0, we diligently recovered sequences by isolating their respective haplotypes from the aforementioned pool of 414 sequences. This meticulous process yielded a total of 34 distinct haplotypes. With the intention of primer design, we harnessed the PRIMER BLAST software package (http://www.ncbi.nlm.nih.gov/tools/primer-blast/) to work on the 34 derived haplotypes. The outcome was an amplicon size uniformly spanning 771 bp across all sequences. To ensure precision, we pinpointed the specific annealing sites for the primers across different *Leishmania* species. This information is visually presented in Additional file 1: Figure S1. Further validation ensued as the designed primers underwent scrutiny through the NCBI BLAST tool software. Moreover, primer physico-chemical attributes, including parameters such as Gibbs free energy (ΔG), melting temperature (Tm), enthalpy (ΔH) and hairpin assessment, underwent thorough evaluation. This assessment was conducted using the online tool Oligoanalyzer 3.1 (https://www.idtdna.com/pages/tools/oligoanalyzer?returnurl=%2Fcalc%2Fanalyzer).

### Analytical sensitivity and specificity of HSP70-Long and HSP70-Short primers

To comprehensively assess the sensitivity and specificity of the primers examined in this study (330-bp HSP70 marker [HSP70-Short] and HSP70-Long), we conducted distinct evaluations: analytical sensitivity, analytical specificity, interference-based specificity and diagnostic performance.

#### Analytical sensitivity

To scale the analytical sensitivity of the HSP70-Long primers, as well as that of the previously published primers, referred to in the following text as HSP70-Short [35], we employed a method involving the determination of the limit of detection (LoD). This method involved conducting a series of sequential DNA dilutions sourced from the reference strain *Leishmania infantum* UA1664 MHOM/CO/87. The dilution series encompassed a spectrum starting from an initial concentration of 1 × 10^6^ parasites/ml and progressively tapering down to a concentration of merely 1 parasite/ml. Subsequently, these diverse dilutions were subjected to amplification using conventional PCR in which both sets of primers (HSP70-Long and HSP70-Short) were employed.

Additionally, as control for sequencing, we prepared mixtures of promastigotes from two different *Leishmania* strains: the reference strain *L. infantum* UA1664 MHOM/CO/87 and *L. amazonensis* strain UA130 [37]. These mixtures consisted of an equal 1:1 ratio of *L. amazonensis* and *L. infantum* at varying concentrations ranging from 1 × 10^6^ to 1 × 10^–1^ parasites/ml. For sequencing purposes, we specifically chose concentrations of 1 × 10^6^ parasites/ml of *L. infantum* and 1 × 10^3^ parasites/ml of *L. amazonensis* (Mix-1), as well as 1 × 10^3^ parasites/ml of *L. infantum* and 1 × 10^6^ parasites/ml of *L. amazonensis* (Mix-2). Both the *L. infantum* and *L. amazonensis* strains were cultivated in Schneider's insect medium supplemented with 10% (v/v) fetal bovine serum. The cultures were maintained at 26 °C and CO_2_ concentration of 5%. The parasites were collected during the late logarithmic growth phase and then subjected to DNA extraction and sequencing.

#### Analytical specificity

The analytical specificity of the HSP70-Short primers were studied in a previous investigation [16] and, therefore, our analysis centered on the HSP70-Long primers. To thoroughly investigate the intra- and interspecies specificity of the HSP70-Long primers, we collected annotation data for *Leishmania* and *Trypanosoma* species which we sourced from the publicly accessible TriTrypDB database (https://tritrypdb.org/common/downloads/Current_Release). Our approach involved the meticulous selection of sequences corresponding to the HSP70 gene for each individual species. Subsequently, each of these sequences per chromosome underwent alignment with the primers that were the subject of our analysis in this study. The sequences obtained were used to build a maximum-likelihood-based phylogenetic tree which we accomplished using FastTree version 2.1.10 Double precision [38]. The robustness of the nodes was rigorously evaluated through the implementation of 1000 bootstrap replicates. The resulting tree was visualized using the Interactive Tree Of Life v4 online tool (http://itol.embl.de) [39]. In addition, we used the Neighbor-Net method in SplitsTree5 [40] to build phylogenetic networks. A Nexus matrix was constructed for haplotype network analysis in Network 3.0 based on a median-joining model with default parameters and 1000 iterations. Finally, we obtained a dissimilarity matrix through sequence alignment using UGENE software. This matrix was then utilized to create a heatmap using the online tool Heatmapper [41]. To construct the heatmap, we employed the average linkage and Pearson distance method.

#### Interference-based specificity

This assay included other trypanosomatids, including *Trypanosoma cruzi* and *Trypanosoma brucei*, as well as a diverse assortment of pathogenic bacteria, fungi and viruses (see Additional file 2: Table S1 for a comprehensive list). DNA from these various pathogens was extracted and provided by the Molecular Microbiology Laboratory (MML) at Mount Sinai Hospital (New York City, NY, USA). These DNA samples were subsequently subjected to amplification via conventional PCR using both sets of primers (HSP70-Long and HSP70-Short).

### Diagnostic performance evaluation with samples collected from hosts with CL and VL

Twenty-seven samples tested in this study, including 26 samples procured from individuals diagnosed with CL. Of these latter 26 samples, 12 were acquired through direct smears of lesions taken from human patients treated at the Dirección de Sanidad Militar, Ejercito Nacional de Colombia in Bogotá; four samples were derived from blood and deposited on FTA cards (3 samples from humans and 1 sample from a domestic dog [*Canis lupus familiaris*]); and 10 samples were extracted from felines (*Felis catus*) residing in various municipalities across Central-Western Venezuela. An additional specimen was examined from a dog in the USA (Texas) exhibiting clinical findings suggestive of VL; this dog was originally from Greece where he presumably was infected.

Samples from felines were collected by carefully scraping lesional tissues and then depositing the scrapings onto FTA classic cards (Whatman Inc., Newton Center, MA, USA). For the case of the dog afflicted with VL, a blood sample was procured by venipuncture using EDTA tubes. This blood sample was meticulously preserved at − 20 °C until the subsequent DNA extraction process was conducted. Additional file 3: Table S2 provides a comprehensive overview of the metadata corresponding to all 27 samples used in the study.

### DNA extraction

Genomic DNA was extracted from serial dilutions of the reference strain *L. infantum* UA1664 MHOM/CO/87, clinical samples and mixtures, using the High Pure PCR Template Preparation Kit (Roche Life Science, Mannheim, Germany) according to the manufacturer's instructions. The ChemagicTM Viral DNA/RNA 300 Kit H96 (CMG-1033-S; PerkinElmer Inc., Waltham, MA, USA) was used for DNA extraction from FTA cards and the blood sample, and the extraction was carried out using the automated ChemagicTM 360 instrument (2024-0020; PerkinElmer Inc.) in accordance with the manufacturer's guidelines. Well-established methods, as previously outlined [42], were applied throughout. Finally, the concentration, quality and integrity of the extracted DNA were evaluated by 1% agarose gel electrophoresis and spectrometry (Nanodrop ND-1000 spectrophotometer; Thermo Fisher Scientific, Waltham, MA, USA).

### PCR amplification

To amplify the four distinct DNA sample types featured in this study, we utilized four specific primers: HSP70-Short-F (5′AGGTGAAGGCGACGAACG 3′) and HSP70-Short-R (5′ CGCTTGTCCATCTTTGCGTC 3′) [35], and HSP70-Long-F (5′-GACTTYCAGGCCAMCATCAC-3′) and HSP70-Long-R (3′-CGAGTACGCGTAGTTCTCCA-5′). These primers were used to amplify: (i) DNA stemming from serial dilutions of the reference strain *L. infantum*, which was integral to the sensitivity assay; (ii) DNA extracted from the mixtures that included both the reference strain *L. infantum* and the *L. amazonensis* strain (UA130); (iii) DNA sourced from clinical samples; and (iv) DNA obtained from other pathogens, a vital component of the specificity assays.

The PCR reaction and cycling conditions for the HSP70-Short primers followed an established protocol reported previously [35]. For the HSP70-Long primers, the reaction mixture was prepared to achieve a final volume of 15 μl (6.25 μl of Platinum Master Mix [Invitrogen, Thermo Fisher Scientific] at 1× concentration, 0.5 μM solution [0.625 μl] of each primer, 2.5 μl of enhancer [Invitrogen, Thermo Fisher Scientific], 3.5 μl of water, 10 ng/μl of DNA template). The thermal cycling profile consisted of an initial denaturation at 94 °C for 5 min (1 cycle); followed by 35 cycles of denaturation (94 °C, 1 min), annealing (52 °C for 30 s) and extension (72 °C for 1 min); and a final extension at 72 °C for 10 min. The quality, integrity and size of the obtained amplicon was assessed by 2% agarose gel electrophoresis, following which the amplicons from each sample were purified using the EXOSAP method (Affymetrix, Santa Clara, CA, USA) following the manufacturer's instructions. Manual assessment of the gels was carried out to identify the presence of a unique band in each case.

### Amplicon-based MinION™ sequencing

The amplicons derived from both clinical samples and mixtures underwent sequencing using the MinION™ platform. To prepare the DNA for this DNA sequencing platform, we performed a series of steps, including end repair, dA tail insertion, barcoding and adapter binding using the EXP-NBD196 kit from Oxford Nanopore Technologies, followed by cleaning of the library using AMPure XP beads (Beckman Coulter Life Sciences, Brea, CA, USA) and finally preparation for loading onto a MinION™ flow cell using the SQK-LSK109 sequencing ligation kit (Oxford Nanopore Technologies). The sequencing process was performed on the miniON platform, utilizing the MinKNOW program (version 20.10.3), in accordance with an established protocol, spanning a 48-h run.

### Bioinformatic analysis

The initial step involved base-calling the raw Fast5 files and demultiplexing the data using the Guppy barcoder. Then, a quality and length filtering process was applied to the reads, which served to eliminate low-quality and potentially chimeric reads. Following filtering, the reads were merged for further analysis. The sequences obtained from HSP70-Long were subjected to comparison against an in-house database constructed during this study, and the sequences derived from HSP70-Short were compared against a previously published database [16]. The taxonomic assignment was conducted locally using BLASTn against the respective databases, with a threshold set at a minimum of 95% identity and an E value of 10. For the analysis, matches exhibiting a relative abundance > 3% were taken into account, a precautionary measure to mitigate potential sequencing errors. Additionally, the relative abundance of *Leishmania* species reads was normalized based on the number of copies of the HSP70 gene present in each species. This adjustment aimed to achieve a more precise estimation of species abundance per individual. Quantitative results were graphically presented using R software version 3.6.1 [16].

## Results

### Analytical sensitivity and specificity of HSP70-Long and HSP70-Short primers

#### Analytical sensitivity

The sensitivity assessment clearly indicated that the HSP70-Long primers possessed a dependable detection capacity of at least 100 parasite-equivalents/ml. In contrast, the HSP70-Short primers showed greater sensitivity, successfully detecting levels as low as 1 equivalent-parasite/ml, as shown in Additional file 4: Figure S2. We also evaluated the capacity of both the HSP70-Long and HSP70-Short primers to identify mixed infections. The outcomes from the assays using the laboratory-designed Mix-1 and Mix-2 samples demonstrated the presence of *L. infantum* and *L. amazonensis* at the anticipated concentrations.

#### Analytical specificity

The specificity of each primer was then assessed in both intra- and interspecies analysis. Since HSP70-Short primers had been evaluated previously [16], we focused on the HSP70-Long primers. The outcomes showed that the HSP70-Long primers annealed to distinct copies of the HSP70 gene within the same *Leishmania* species, thereby demonstrating a notable level of intraspecies specificity.

**Fig. 1:**
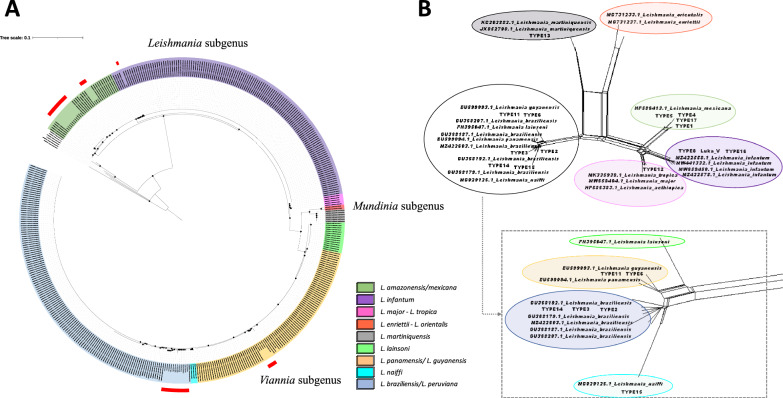
Phylogenetic analysis based on HSP70-Long sequences used for the reference database. **A** The tree represents the phylogenetic analysis based on 414 HSP70-Long sequences used for the reference database and the 27 sequences included in this study. The colors of the inner circle represent the different *Leishmania* species, and the red color of the external circle indicates the samples analyzed in this study. Black dots represent well-supported nodes (Bootstrap ≥ 90). **B** Phylogenetic network (Neighbor-Net) constructed in SplitsTree 5, based on 34 haplotypes from HSP70-Long reference sequences. The square shows enlargements of the clusters represented by *Viannia* subgenera species. HSP70-Long, 771-bp 70-kDa heat shock protein gene-based marker

Furthermore, these primers showcased a robust ability to discriminate between various *Leishmania* species, as illustrated in Additional file 5: Figure S3. To assess the ability of the HSP70-Long primers to differentiate between various *Leishmania* species, we conducted an in-depth analysis of their discriminatory potential. Phylogenetic analysis of the acquired sequences revealed three distinct, well-supported clusters, (bootstrap values of ≥ 90.0), with the first cluster associated with *Leishmania* subgenus species, the second cluster associated with *Mundinia* subgenus species and the third cluster represented by species belonging to the *Viannia* subgenus (Fig. 1a). Within the *Leishmania* subgenus cluster, three distinct groups were identified, with the first group (Fig. 1a, highlighted in green) encompassing sequences belonging to the *L. mexicana* complex, the second group (Fig. 1a, highlighted in purple) consisting of sequences from *L. infantum* and the third group (Fig. 1a, highlighted in fuchsia) comprising sequences from the *Leishmania major* and *Leishmania tropica* complexes*.*

For the *Mundinia* subgenus, two distinct groups were discerned. The first group (Fig. 1a, highlighted in light red) encompassed sequences associated with *Leishmania enrietti* and *Leishmania orientalis*, and the second group (Fig. 1a, highlighted in gray) comprised sequences from *Leishmania martiquinensis* (Fig. 1a).

Within the *Viannia* subgenus, four distinct groups were identified. The first group (Fig. 1a, highlighted in lime) included sequences of *Leishmania lainsoni*, the second group (Fig. 1a, highlighted in light yellow) comprised sequences from the *Leishmania guyanensis* complex, the third group (Fig. 1a, highlighted in cyan) was composed of sequences from *Leishmania naiffi* and the fourth group (Fig. 1a, highlighted in light blue) featured sequences from the *Leishmania braziliensis* complex species (Fig. 1a). These findings were further validated by the phylogenetic tree topologies obtained through SplitsTree5 (Fig. 1b). Finally, the haplotype network showcased 34 distinct sequences (Additional file 6: Figure S4), underscoring the robust discriminatory prowess of the designed primers.

#### Interference-based specificity

The outcomes of assays utilizing the HSP70-Short primers revealed amplification solely for *T. cruzi* DNA, with no observable amplification detected for any other DNA tested. In contrast, the outcomes of assays employing the HSP70-Long primers demonstrated a consistent absence of amplification for all the tested DNA samples.

### Reads analysis by HSP70 amplicon-based MinION™ sequencing

This analysis focused on the reads acquired following the MinKNOW platform's implementation of reading filters for quality and length. The outcomes of the HSP70-Short amplicon-based MinION™ sequencing revealed a spectrum of reads ranging from 947 to 678,302, and those of the HSP70-Long amplicon-based MinION™ sequencing yielded a range of reads from 137 to 204,869 (see Additional file 7: Table S3).

### Diagnostic performance

By employing gene amplicon-based NGS targeting the HSP70-Long and HSP70-Short genes, we not only successfully detected a range of *Leishmania* species within the human, feline and canine samples but also established a strong agreement between the two primer sets studied. Infection events were recognized in 85% (*n* = 23/27) of the analyzed samples, while coinfection events were noted in the remaining 15% (*n* = 4) of samples.

**Fig. 2:**
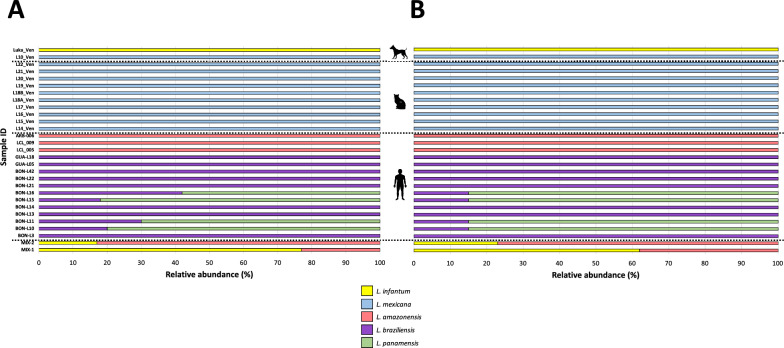
Relative abundances of *Leishmania* species identified in the samples analyzed.** A**,** B** Relative abundance of *Leishmania* species identified in the samples analyzed using HSP70-Long MinION™ sequencing (**A**) and HSP70-Short MinION™ sequencing (**B**). Mix refers to the mixtures of *L. infantum* and *L. amazonensis* made in the laboratory: Mix-1 contained 1 × 10^6^ parasites/ml of *L. infantum* and 1 × 10^3^ parasites/ml of *L. amazonensis*; Mix-2 contained 1 × 10^3^ parasites/ml of *L. infantum* and 1 × 10^6^ parasites/ml of *L. amazonensis*. Color bars represent the *Leishmania* species found in the sample, HSP70-Long, 771-bp 70-kDa heat shock protein gene-based marker; HSP70-Short, previously published heat shock protein gene-based marker spanning 330 bp.

Among the detected species, *L. braziliensis* emerged as the predominant species in the human samples collected from the Colombian army. In comparison, in samples from Venezuela, *L. amazonensis* emerged as the prevailing *Leishmania* species and, notably, *L. mexicana* was predominant present in the feline samples while *L. infantum* was exclusively detected in the canine sample affected by VL. The distinct distributions of these across different host populations are graphically represented in Fig. 2.

We also identified double coinfection events between *Leishmania panamensis* and *L. braziliensis* (Fig. 2a; Additional file 8: Table S4). These findings were in agreement with those obtained for the HSP70-Short gene amplicon-based NGS (Fig. 2b). The most relevant difference was in the relative species abundance identified in two analyzed samples. For example, in the case of sample BON-L11, the relative proportion between *L. braziliensis* and *L. panamensis* was 30/70 with the HSP70-Long approach, and 15/85 with the HSP70-Short approach. A similar pattern emerged in the BON-L16 sample, where the relative abundances were 42/58 (HSP70-Long) and 15/85 (HSP70-Short) for *L. braziliensis* and *L. panamensis*, respectively (Fig. 2).

Subsequently, we aimed to evaluate the intraspecies genetic variability by analyzing the clinical samples collected during this study. The outcomes revealed a moderate level of nucleotide diversity within *L. braziliensis*. Among the 12 sequences analyzed, we identified two different clusters. A total of five polymorphic sites and three distinct haplotypes were identified. The dissimilarity heatmap further illustrated the differentiation between these clusters (Additional file 9: Figure S5).

## Discussion

Numerous studies performed to date have highlighted the potential of the HSP70 marker to distinguish various *Leishmania* species [43–47]. This marker has also been instrumental in reconstructing phylogenetic relationships and establishing taxonomy classifications [27, 29]. Such studies have employed diverse molecular techniques and sequencing methods. Notably, some studies have leveraged the HSP70-based NGS to distinguish mixtures of sequences within a single sample [16, 17, 48], while others have effectively employed the great accuracy of the MinION™ long-read sequencing technology to detect and identify *Leishmania* species in different clinical forms [15].

However, many of these earlier studies used short markers, which could potentially limit the ability to capture nucleotide-level differences among different *Leishmania* species. In the present study, we report the design of a large marker of 771 bp based on *hsp70* (HSP70-Long) and the results of a pioneering evaluation assessment of its sensitivity, specificity and diagnostic prowess to identify diverse *Leishmania* species using the MinION™ sequencing technology. We also undertook a comparative analysis, pitting this newly designed marker against a previously established one (HSP70-Short) [35] in a comparison of performance.

The findings of the present study underscore that the HSP70-Short PCR marker exhibited the highest sensitivity in detecting *L. infantum* DNA, achieving a remarkable LoD as low as 1 parasite-equivalent/ml. In contrast, the HSP70-Long PCR marker showed a higher LoD of 1 × 10^2^ parasite-equivalents/ml. These findings are in agreement with prior research that consistently emphasized the heightened sensitivity of shorter segments of the HSP70 gene [49, 50]. The enhanced sensitivity of the short fragment likely accounts for its superior performance in detecting *L. infantum* DNA, as revealed in our study. Incorporating this shorter fragment into PCR assays has the potential to substantially enhance the precision and dependability of *Leishmania* species detection.

When comparing the LoD achieved for the HSP70-Short marker with findings from earlier reports, we noted distinct differences. Notably, our results contrast with those of Leon et al., who previously documented an LoD of 1 × 10^1^ parasite-equivalents/ml [51]. Similarly, the LoD reported by Filgueira et al. was 10^2^ parasite-equivalents/reaction [52]. This contrast in LoD values might be explained by the specific *Leishmania* species being studied. It is important to highlight that while the LOD in the present study was determined specifically for *L. infantum*, this value could exhibit variations for other species due to the copy number of the HSP70 gene, which varies depending on the *Leishmania* subgenera analyzed [50, 53, 54].

When we compared the LoD achieved with conventional PCR with those from other *Leishmania* species detection and quantification techniques, particularly those targeting the HSP70 gene, such as qPCR and PCR-RFLP, we noted significant differences. Various studies have outlined the range of LoDs obtained by qPCR LoDs which LoDs ranging from 1 × 10^–1^ parasite-equivalents/ml to 10 parasite-equivalents/ml [35, 51, 52]. In contrast, previous investigations centered around PCR–RFLP have shown a remarkable increase in sensitivity, with a detection capability ranging from 0.1 ng to 100 fg of DNA [49, 50]. These findings are extremely relevant in the context of analyzing clinical and biological samples, where the sensitivity of the PCRs is crucial due to samples often containing a small number of parasites.

In terms of primer specificity, our observations revealed that the HSP70-Short primers were not exclusive to *Leishmania* DNA, as they also amplified *T. cruzi* DNA. This cross-reactivity indicates a potential limitation in the application of these primers for precise *Leishmania* detection. In contrast, the HSP70-Long primers demonstrated complete exclusivity in detecting *Leishmania* DNA without any interference from the DNA of other trypanosomatids or other non-target organisms. This remarkable specificity enhances the reliability and accuracy of HSP70-Long primers for specific *Leishmania* identification, making them a valuable tool for diagnostic applications and research purposes. Taking into account the co-endemism of *T. cruzi* and *Leishmania* in many regions, infecting both animals [55, 56] and humans [57, 58], emphasis must be placed on the importance of using genus-specific PCRs to avoid misdiagnosis or missed detection of mixed infections involving both *T. cruzi* and *Leishmania*. By utilizing the HSP70-Long primers, which are designed exclusively for *Leishmania* species, it is possible to effectively tackle this concern and achieve more dependable outcomes, particularly in regions endemic for leishmaniasis. Nonetheless, it is worth noting that this marker might not be efficacious in areas with a high incidence of Chagas Disease or in regions where both parasites coexist sympatrically. In such instances, the HSP70-Short marker could certainly provide higher resolution, given its capability to reliably differentiate among various trypanosomatids and consistently detect coinfection events within a single host [16, 59].

To assess clinical performance, we conducted MinION™ sequencing using both HSP70-Short and HSP70-Long markers on samples from both humans and animals. The outcomes from human samples consistently indicated two significant findings. Firstly *L. braziliensis* emerged as the predominant *Leishmania* species within the Colombian military population. Secondly, exclusive detection of *L. amazonensis* occurred in samples collected from central-western Venezuela (Figs. 1 and 2). Despite the relatively limited sample size scrutinized in this study, our results concur with earlier studies [43, 60–62]. These results underline not only the prevalence of these species in vulnerable populations that frequently deploy to high-endemic regions, exhibiting continuous movement between urban and rural areas, where numerous vectors and reservoirs coexist, thereby contributing to the spread of leishmaniasis [63, 64], but also spotlight the predominance of species linked to different forms of Leishmaniasis [65–68], which exhibit a notable capacity to induce a low immune response [68, 69] while displaying variable response to anti-leishmanial therapy [68, 70].

Our findings revealed the dominance of *L. mexicana* as the primary infecting species in the feline samples (Figs. 1, 2). These results are in agreement with the findings obtained from studies conducted in Texas (USA) [71, 72] as well as observations reported in Venezuela [73–75]. In these regions, *L. mexicana* has been identified as the primary causative agent responsible for feline leishmaniasis. This stands in contrast to observations made in the Old World where *L. infantum*, *L. tropica* and *L. major* are the most prevalent species associated with this disease. The active circulation of *L. mexicana* in cats carries significant clinical implications for the health of the infected animal due to the diverse range of clinical lesions it causes (such as ulcers, verrucous lesions, papules and scaly plaques) and the limited availability of preventive measures and therapeutic options for treating feline leishmaniasis [75]. It also has repercussions for human and canine health, given the ongoing coexistence of cats and humans and the potential role of cats as potential sentinel reservoir hosts in endemic areas of zoonotic VL [76]. Given the widespread occurrence of feline leishmaniasis globally, spanning regions in Europe, the Middle East (Egypt, Iran and Turkey), Thailand, Texas (USA) [77, 78], as well as several countries across the Americas (Mexico, Brazil and Venezuela) [73, 79], it is imperative to emphasize the need to include leishmaniasis in the differential diagnosis of cutaneous lesions for both dogs and cats.

On the other hand, HSP70-based MinION™ sequencing revealed coinfection events in 15% of the CL samples analyzed, all of which were attributed to *L. braziliensis/L. panamensis* coinfection (Fig. 2a). These findings consistently align with those obtained with the HSP70-Short marker (Fig. 2b; Additional file 8: Table S4). The detection of coinfection events underscores the importance of accurately identifying the responsible *Leishmania* species in infections. Such events can significantly impact the natural course of the leishmaniasis, as previously documented [80], and add complexity to both the diagnosis of and therapeutic approach to the disease.

Moreover, these findings revealed that coinfections events are more prevalent than previously thought. Coinfection is not limited solely to humans, as indicated by our current study and previous research [16, 17, 34, 80, 81], but is also evident in sand fly vectors [16, 17, 82] and reservoirs [16, 17, 83, 84]. The identification of these coinfection events holds paramount importance in comprehending the intricate eco-epidemiology of the disease. Furthermore, this knowledge assumes a pivotal role in shaping novel and enhanced strategies for the control, prevention and diagnosis of Leishmaniasis.

Both this study and earlier research demonstrated that HSP70 markers are a reliable and efficient molecular approach not only in screening for the presence of *Leishmania* species, but also for assessing their inter- and intraspecies genetic diversity [16]. The HSP70-Long primers analyzed in this study exhibited a robust ability to discriminate between different species within the *Leishmania* genus (Fig. 1). Furthermore, this marker facilitated the identification of distinct haplotypes within the analyzed sequences of *L. braziliensis* (Additional file 9: Fig. S5), further confirming the substantial intraspecific genetic diversity within this species—diversity that might contribute to its survival and adaptation to various ecological niches.

There are a number of limitations to this study. First, the number of samples analyzed was relatively small number, particularly those associated with VL. Second, the geographical distribution of the samples evaluated was limited, with the main focus on regions where co-circulation of different *Leishmania* species exists [43, 85] and the inclusion of more *Leishmania* species to analyze the sensitivity of both primer sets. Therefore, future studies with more extensive sampling should be conducted to further enhance our understanding.

## Conclusions

In conclusion, this study is the first to demonstrate the value of HSP70-based MinION™ sequencing as a potent technology for not only identifying and distinguishing *Leishmania* species but also for detecting mixed infections within clinical samples. This technology stands out for its exceptional specificity and cost-effectiveness, when compared to conventional sequencing platforms, as previously demonstrated [15–17, 86]. In contrast with NGS Illumina sequencing, the MinION™ DNA sequencing system from Oxford Nanopore Technologies offers the advantage of remarkably low sequencing costs (approximately US$26 per sample vs. US$100 with paired-end short read technology) and a streamlined preparation process, and also eliminates the need for extensive equipment maintenance. Moreover, MinION™ presents operational time benefits, which have been documented to be as short as 6 h from sample collection [20, 87]. This system also capitalizes on the benefit of generating long reads [88]. It is crucial to emphasize that this study was designed for validation purposes, with a sample size we believe is well-suited for this objective (it is recommended to have 20–30 samples for validation purposes in the literature). It is also worth noting that obtaining the required number of samples to meet the objective of this study was a challenging endeavor. Finally, we anticipate that future epidemiological studies will included larger sample sizes and provide important information on *Leishmania* species worldwide. Despite the excellent specificity of HSP70-Long primers, this study revealed their lower sensitivity compared to HSP70-Short primers, thereby highlighting the trade-off between sensitivity levels in the two primer sets, with the HSP70-Short primers exhibiting superior performance in detecting lower parasite concentrations.

### Supplementary Information

**Additional file 1: Figure S1.** Multiple alignment of 34 haplotypes from HSP70-Long reference sequences and annealing sites of primers. **A** Annealing sites for forward primer, **B** annealing sites for reverse primer.

**Additional file 2: Table S1.** Panel of organisms used in the specificity assay, including bacteria, fungi, viruses and parasites.

**Additional file 3: Table S2.** Metadata of samples included in the study.

**Additional file 4: Figure S2.** Analytical sensitivity analysis*.* The figure illustrates the results of conventional PCR amplification, initiated at a concentration of 1 × 10^6^ parasites/ml and reducing to 1 × 10^–1^ parasite/m, employing HSP70-Long (**A**) and HSP70-Short (**B**) primers. Line 1, PPM; lines 2–9, 1 × 10^6^ to 1 × 10^–1^ parasites/ml; line 10, negative control.

**Additional file 5: Figure S3.** Phylogenetic relationship between the different HSP70 gene copies in trypanosomatids. The figure represents the phylogenetic analysis of HSP70-Long sequences in trypanosomatids, as recognized by the primers used in the study. The black dots represent well-supported nodes (Bootstrap ≥ 90).

**Additional file 6: Figure S4.** Network analysis of 34 haplotypes from HSP70-Long reference sequences. Each sequenced haplotype is represented by a circle. Black lines on the branches indicate the mutational changes between the different haplotypes.

**Additional file 7: Table S3.** Number of reads obtained from HSP70-Long- and HSP70-Short amplicon-based MinION™ sequencing for each sample included in the study

**Additional file 8: Table S4.** Comparison of the results obtained between the HSP70-Long- and HSP70-Short amplicon-based MinION™ sequencing, in each sample included in the study.

**Additional file 9: Figure S5.** Analysis of intraspecies diversity among *L. braziliensis* sequences. **A** Phylogenetic relationship among *L. braziliensis hsp70* sequences. **B** Haplotype of *L. braziliensis* network based on *hsp70* sequences. Each haplotype is denoted by a circle and mutational steps between haplotypes are depicted by the number of lines. **C** Heatmap illustrating pairwise comparison of *L. braziliensis* sequences.

## Data Availability

The dataset generated during the study was deposited at the European Nucleotide Archive (ENA) under the bioproject code PRJNA855474.
