# Supplementary material for: Validation of Oxford nanopore sequencing for improved New World Leishmania species identification via analysis of 70-kDA heat shock protein
Source: Parasit Vectors. 2023 Dec 18;16:458. doi: 10.1186/s13071-023-06073-9 (PMC10726620; doi:10.1186/s13071-023-06073-9)
Supplement: Supplementary file 1 — Additional file 1: Figure S1. Multiple alignment of 34 haplotypes from HSP70-Long reference sequences and annealing sites of primers. A Annealing sites for forward primer, B annealing sites for reverse primer. [file 13071_2023_6073_MOESM1_ESM.pdf]

A

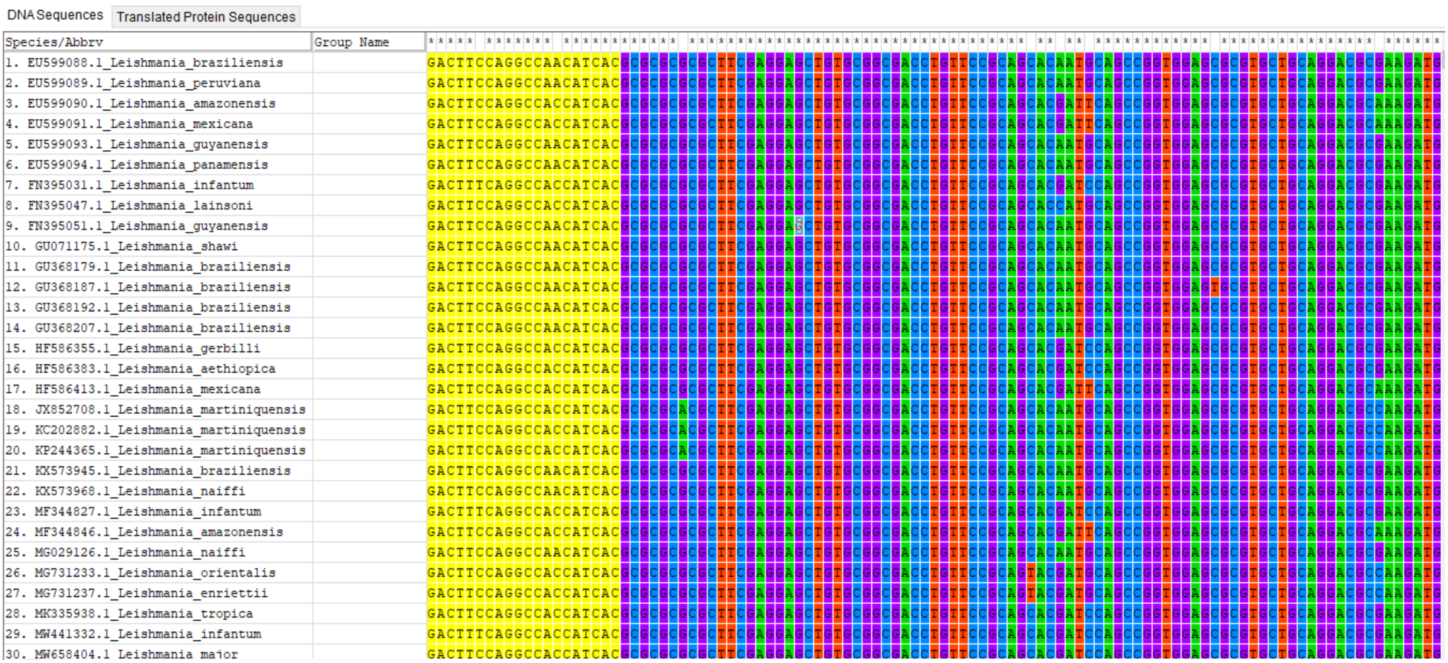
DNA Sequences
Translated Protein Sequences
Species/Abbrv
Group Name
1. EU599088.1_Leishmania_braziliensis
2. EU599089.1_Leishmania_peruviana
3. EU599090.1_Leishmania_amazonensis
4. EU599091.1_Leishmania_mexicana
5. EU599093.1_Leishmania_guyanensis
6. EU599094.1_Leishmania_panamensis
7. FN395031.1_Leishmania_infantum
8. FN395047.1_Leishmania_lainsoni
9. FN395051.1_Leishmania_guyanensis
10. GU071175.1_Leishmania_shawi
11. GU368179.1_Leishmania_braziliensis
12. GU368187.1_Leishmania_braziliensis
13. GU368192.1_Leishmania_braziliensis
14. GU368207.1_Leishmania_braziliensis
15. HF586355.1_Leishmania_gerbilli
16. HF586383.1_Leishmania_aethiopica
17. HF586413.1_Leishmania_mexicana
18. JX852708.1_Leishmania_martiniquensis
19. KC202882.1_Leishmania_martiniquensis
20. KP244365.1_Leishmania_martiniquensis
21. KX573945.1_Leishmania_braziliensis
22. KX573968.1_Leishmania_naiffi
23. MF344827.1_Leishmania_infantum
24. MF344846.1_Leishmania_amazonensis
25. MG029126.1_Leishmania_naiffi
26. MG731233.1_Leishmania_orientalis
27. MG731237.1_Leishmania_enriettii
28. MK335938.1_Leishmania_tropica
29. MW441332.1_Leishmania_infantum
30. MW658404.1_Leishmania_major

B

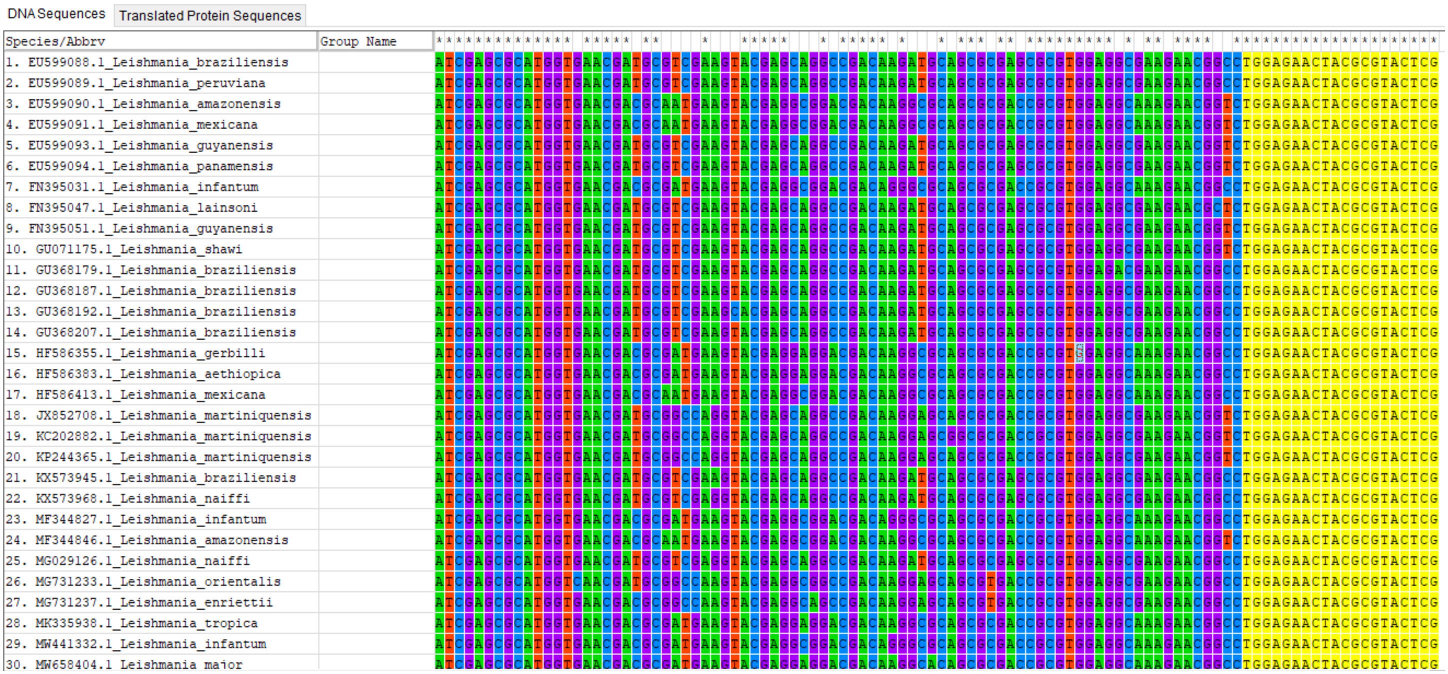
DNA Sequences
Translated Protein Sequences
Species/Abbrv
Group Name
1. EU599088.1_Leishmania_braziliensis
2. EU599089.1_Leishmania_peruviana
3. EU599090.1_Leishmania_amazonensis
4. EU599091.1_Leishmania_mexicana
5. EU599093.1_Leishmania_guyanensis
6. EU599094.1_Leishmania_panamensis
7. FN395031.1_Leishmania_infantum
8. FN395047.1_Leishmania_lainsoni
9. FN395051.1_Leishmania_guyanensis
10. GU071175.1_Leishmania_shawi
11. GU368179.1_Leishmania_braziliensis
12. GU368187.1_Leishmania_braziliensis
13. GU368192.1_Leishmania_braziliensis
14. GU368207.1_Leishmania_braziliensis
15. HF586355.1_Leishmania_gerbilli
16. HF586383.1_Leishmania_aethiopica
17. HF586413.1_Leishmania_mexicana
18. JX852708.1_Leishmania_martiniquensis
19. KC202882.1_Leishmania_martiniquensis
20. KP244365.1_Leishmania_martiniquensis
21. KX573945.1_Leishmania_braziliensis
22. KX573968.1_Leishmania_naiffi
23. MF344827.1_Leishmania_infantum
24. MF344846.1_Leishmania_amazonensis
25. MG029126.1_Leishmania_naiffi
26. MG731233.1_Leishmania_orientalis
27. MG731237.1_Leishmania_enriettii
28. MK335938.1_Leishmania_tropica
29. MW441332.1_Leishmania_infantum
30. MW658404.1_Leishmania_major
