# Supplementary material for: Validation of Oxford nanopore sequencing for improved New World Leishmania species identification via analysis of 70-kDA heat shock protein
Source: Parasit Vectors. 2023 Dec 18;16:458. doi: 10.1186/s13071-023-06073-9 (PMC10726620; doi:10.1186/s13071-023-06073-9)
Supplement: Supplementary file 2 — Additional file 2: Table S1. Panel of organisms used in the specificity assay, including bacteria, fungi, viruses and parasites. [file 13071_2023_6073_MOESM2_ESM.docx]

**Table S1.** Panel of organisms used in the specificity assay. Including bacteria, fungi, viruses and parasites.

|  | | | | | | | | | | | | | | | | |
| --- | --- | --- | --- | --- | --- | --- | --- | --- | --- | --- | --- | --- | --- | --- | --- | --- |
| **Organism** |  |  |  |  |  |  |  |  |  |  |  |  |  |  |  |  |
| *Bacillus cereus group* |  |  |  |  |  |  |  |  |  |  |  |  |  |  |  |  |
| *Cutibacterium acnes* |  |  |  |  |  |  |  |  |  |  |  |  |  |  |  |  |
| *Enterococcus durian atcc 11576* |  |  |  |  |  |  |  |  |  |  |  |  |  |  |  |  |
| *Listeria monocytogenes* |  |  |  |  |  |  |  |  |  |  |  |  |  |  |  |  |
| *Micrococcus luteus* |  |  |  |  |  |  |  |  |  |  |  |  |  |  |  |  |
| *Staphylococcus epidermidis atcc 12228* |  |  |  |  |  |  |  |  |  |  |  |  |  |  |  |  |
| *Staphylococcus aureus atcc 29213 (OSSA)* |  |  |  |  |  |  |  |  |  |  |  |  |  |  |  |  |
| *Staphylococcus aureus atcc 25923* |  |  |  |  |  |  |  |  |  |  |  |  |  |  |  |  |
| *Streptococcus mitis/oralis* |  |  |  |  |  |  |  |  |  |  |  |  |  |  |  |  |
| *Streptococcus pneumoniae atcc 49619* |  |  |  |  |  |  |  |  |  |  |  |  |  |  |  |  |
| *Acinetobacter baumannii* |  |  |  |  |  |  |  |  |  |  |  |  |  |  |  |  |
| *Bacteroides fragilis* |  |  |  |  |  |  |  |  |  |  |  |  |  |  |  |  |
| *Enterobacter cloacae subsp. cloacae atcc 13047* |  |  |  |  |  |  |  |  |  |  |  |  |  |  |  |  |
| *Escherichia coli atcc 35218* |  |  |  |  |  |  |  |  |  |  |  |  |  |  |  |  |
| *Haemophilus influenzae atcc 10211* |  |  |  |  |  |  |  |  |  |  |  |  |  |  |  |  |
| *Klebsiella oxytoca atcc 49131* |  |  |  |  |  |  |  |  |  |  |  |  |  |  |  |  |
| *Morganella morganii* |  |  |  |  |  |  |  |  |  |  |  |  |  |  |  |  |
| *Neisseria meningitidis atcc 13090* |  |  |  |  |  |  |  |  |  |  |  |  |  |  |  |  |
| *Candida glabrata atcc 15126* |  |  |  |  |  |  |  |  |  |  |  |  |  |  |  |  |
| *Candida tropicalis atcc 9968* |  |  |  |  |  |  |  |  |  |  |  |  |  |  |  |  |
| *Candida albicans atcc 60193* |  |  |  |  |  |  |  |  |  |  |  |  |  |  |  |  |
| *Human T-lymphotropic virus type 1* |  |  |  |  |  |  |  |  |  |  |  |  |  |  |  |  |
| *Human Papillomavirus-16* |  |  |  |  |  |  |  |  |  |  |  |  |  |  |  |  |
| *Human Immunodeficiency Virus* |  |  |  |  |  |  |  |  |  |  |  |  |  |  |  |  |
| *Hepatitis C virus* |  |  |  |  |  |  |  |  |  |  |  |  |  |  |  |  |
| *Trypanosoma cruzi* |  |  |  |  |  |  |  |  |  |  |  |  |  |  |  |  |
| *Trypanosoma brucei* |  |  |  |  |  |  |  |  |  |  |  |  |  |  |  |  |
