# Supplementary material for: Validation of Oxford nanopore sequencing for improved New World Leishmania species identification via analysis of 70-kDA heat shock protein
Source: Parasit Vectors. 2023 Dec 18;16:458. doi: 10.1186/s13071-023-06073-9 (PMC10726620; doi:10.1186/s13071-023-06073-9)
Supplement: Supplementary file 3 — Additional file 3: Table S2. Metadata of samples included in the study. [file 13071_2023_6073_MOESM3_ESM.docx]

**Table S2.** Metadata of samples included in the study.

| **CL Patients** | | | | | | | | | | | | | | | | |
| --- | --- | --- | --- | --- | --- | --- | --- | --- | --- | --- | --- | --- | --- | --- | --- | --- |
| **Sample ID** | **Age (Years)** | **Sampling Date** | **Occupation** | **Militar Grade** | **Country** | **Department** | **Municipality** | **Possible site of infection** | | **Previous CL** | **Previous ML** | **Recurring injury** | **Daily repellent use** | **Anatomical location of the lesions** | **Concomitant diseases** | **Topical medication** |
|  |  |  |  |  |  |  |  | **Department** | **Municipality** |  |  |  |  |  |  |  |
| BON-L3 | ND | 19/04/2022 | Militar | Professional soldier | Colombia | Caquetá | Cartagena de Chaira | ND | ND | NO | NO | NO | YES | ARM | NO | NO |
| BON-L10 | 19 | 09/06/2022 | Militar | Regular Soldier | Colombia | Antioquia | Ituango | Antioquia | Ituango | NO | NO | NO | NO | HAND | NO | NO |
| BON-L11 | 19 | 09/06/2022 | Militar | Regular Soldier | Colombia | Antioquia | Bello | Antioquia | Puerto Valdivia | NO | NO | NO | NO | FOREARM | NO | NO |
| BON-L13 | 24 | 19/04/2022 | Militar | Professional soldier | Colombia | Caquetá | Cartagena de Chaira | Caquetá | San Vicente del Caguan | NO | NO | NO | NO | ARM | NO | CLOTRIMAZOL |
| BON-L14 | 27 | 30/06/2022 | Militar | Aircraftman | Colombia | Putumayo | Puerto Asis | Putumayo | Puerto Asis | NO | NO | NO | YES | HAND | NO | NO |
| BON-L15 | 24 | 30/06/2022 | Militar | Regular Soldier | Colombia | Sántander | Simitarra | Sántander | Simitarra | NO | NO | NO | NO | FOREARM | NO | NO |
| BON-L16 | 21 | 11/07/2022 | Militar | Regular Soldier | Colombia | Putumayo | Villa Garzón | Cauca | Piamonte | NO | NO | NO | YES | HAND | NO | NO |
| BON-L21 | 32 | 11/07/2022 | Militar | Professional soldier | Colombia | Meta | Macarena | Meta | Macarena | YES | YES | YES | YES | LEG | NO | NO |
| BON-L22 | 22 | 11/07/2022 | Militar | Professional soldier | Colombia | Tolima | Melgar | Meta | Macarena | NO | NO | NO | YES | ARM | NO | NO |
| BON -L42 | 32 | 06/10/2022 | Militar | Professional soldier | Colombia | Caquetá | San Vicente del Caguan | Caquetá | San Vicente del Caguan | NO | NO | NO | YES | WRIST | NO | ND |
| GUA-L05 | 29 | 06/01/2022 | Militar | ND | Colombia | Guaviare | San José del Guaviare | ND | ND | NO | NO | NO | YES | ND | NO | NO |
| GUA-L18 | 19 | 05/07/2022 | Militar | Regular Soldier | Colombia | Guaviare | San José del Guaviare | Guaviare | San José del Guaviare | NO | NO | NO | NO | WRIST | NO | NO |
| LCL_005 | 45 | ND | ND | ND | Venezuela | ND | ND | ND | ND | ND | ND | ND | ND | ND | ND | ND |
| LCL_009 | 29 | ND | ND | ND | Venezuela | ND | ND | ND | ND | ND | ND | ND | ND | ND | ND | ND |
| ARB_006 | ND | ND | ND | ND | Venezuela | ND | ND | ND | ND | ND | ND | ND | ND | ND | ND | ND |

| **Reservoirs** | | | | | | | | | | | |  |
| --- | --- | --- | --- | --- | --- | --- | --- | --- | --- | --- | --- | --- |
| **Sample ID** | **Asociation** | **Sample origen** | **Country** | **State** | **City** | **Location** | **Shape of lesion** | **Anatomical location** | **Disease Evolution** | **Sampling date** | **Treatment** |  |
| L14_Ven | Domestic Reservoir | *Felis catus* | Venezuela | Lara | Barquisimeto | Santa Elena | Nodular | Nose | 12 | February 2022 | ND |  |
| L15_Ven | Domestic Reservoir | *Felis catus* | Venezuela | Lara | Barquisimeto | El Manzano | Nodular | Nose | 6 | February 2022 | ND |  |
| L16_Ven | Domestic Reservoir | *Felis catus* | Venezuela | Lara | Barquisimeto | El Manzano | Nodular | Nose | 2 | February 2022 | ND |  |
| L17_Ven | Domestic Reservoir | *Felis catus* | Venezuela | Lara | Barquisimeto | El Manzano | Ulcerative | Nose | ND | February 2022 | ND |  |
| L18A_Ven | Domestic Reservoir | *Felis catus* | Venezuela | Lara | Barquisimeto | El Manzano | Nodular | Nose | 8 | February 2022 | ND |  |
| L18B_Ven | Domestic Reservoir | *Felis catus* | Venezuela | Lara | Barquisimeto | El Manzano | Nodular | Ear | 8 | February 2022 | ND |  |
| L19_Ven | Domestic Reservoir | *Felis catus* | Venezuela | Lara | Barquisimeto | Urb del este | Nodular/Ulcerative | Nose | 15 | March 2022 | cryosurgery |  |
| L20_Ven | Domestic Reservoir | *Felis catus* | Venezuela | Lara | Barquisimeto | Urb del este | Nodular/Ulcerative | Nose | 15 | March 2022 | cryosurgery |  |
| L21_Ven | Domestic Reservoir | *Felis catus* | Venezuela | Lara | Barquisimeto | La Ruezga Norte | Nodular/Ulcerative | Nose and Ear | 50 | June 2020 | Levofloxacine/Itraconazole |  |
| L22_Ven | Domestic Reservoir | *Felis catus* | Venezuela | Lara | Barquisimeto | Concha acústica | Nodular/Ulcerative | Nose | 24 | November 2021 | ND |  |
| L10_Ven | Domestic Reservoir | *Canis lupus familiaris* | Venezuela | Lara | Barquisimeto | Cadubare | Ulcerative | Nose | 6 | August 202 | Levofloxacine/Itraconazole |  |
| Luka | Domestic Reservoir | *Canis lupus familiaris* | USA | ND | Texas | ND | Visceral | ND | ND | ND | ND |  |

ND: No Dat
