# Supplementary figures and images for: Validation of Oxford nanopore sequencing for improved New World Leishmania species identification via analysis of 70-kDA heat shock protein
Source: Parasit Vectors. 2023 Dec 18;16:458. doi: 10.1186/s13071-023-06073-9 (PMC10726620; doi:10.1186/s13071-023-06073-9)

**A**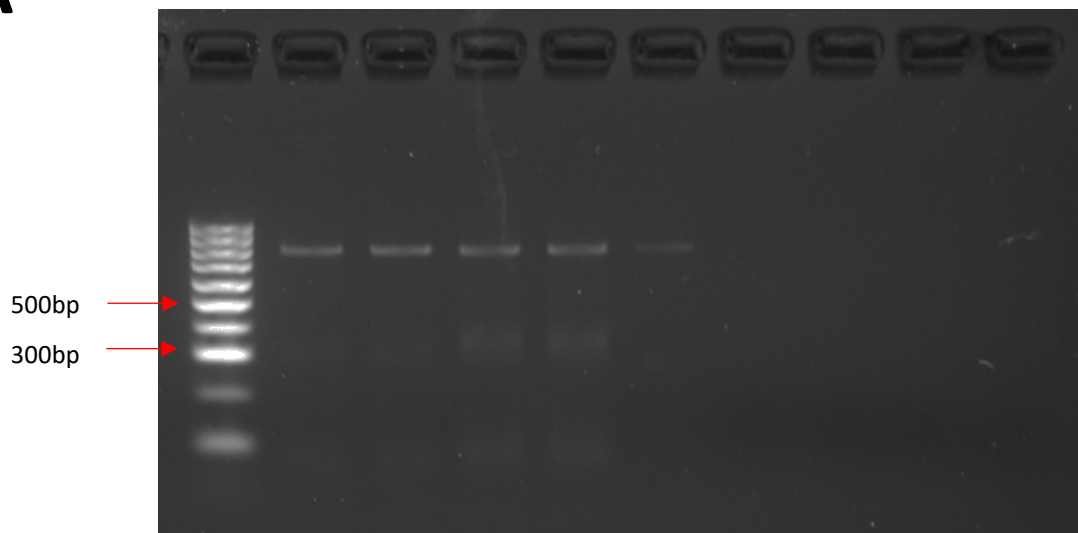**B**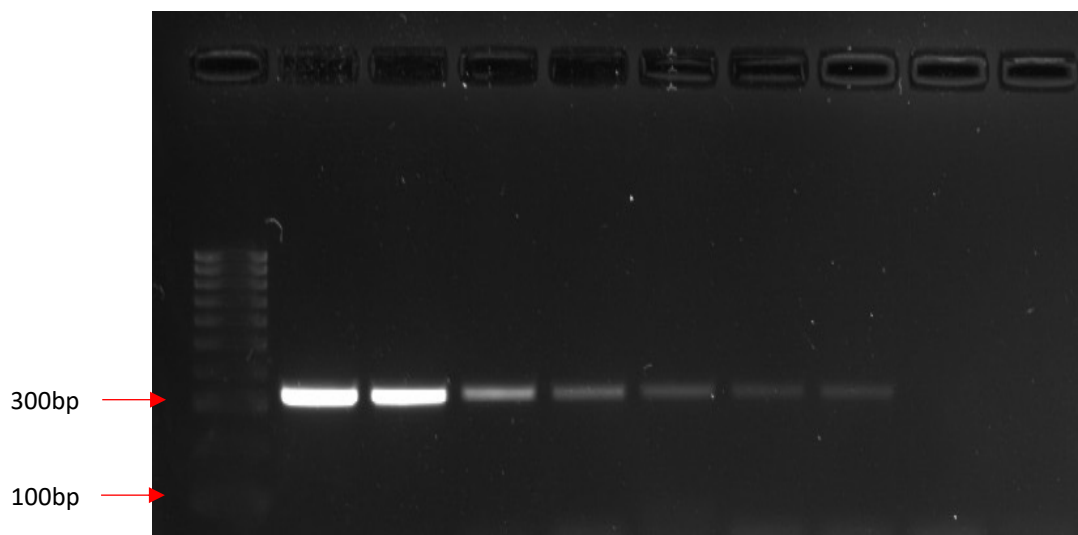

Supplement: Supplementary file 4 — Additional file 4: Figure S2. Analytical sensitivity analysis. The figure illustrates the results of conventional PCR amplification, initiated at a concentration of 1 × 10^6^ parasites/ml and reducing to 1 × 10^–1^ parasite/m, employing HSP70-Long (A) and HSP70-Short (B) primers. Line 1, PPM; lines 2–9, 1 × 10^6^ to 1 × 10^–1^ parasites/ml; line 10, negative control. [file 13071_2023_6073_MOESM4_ESM.pdf]

Tree scale: 0.01

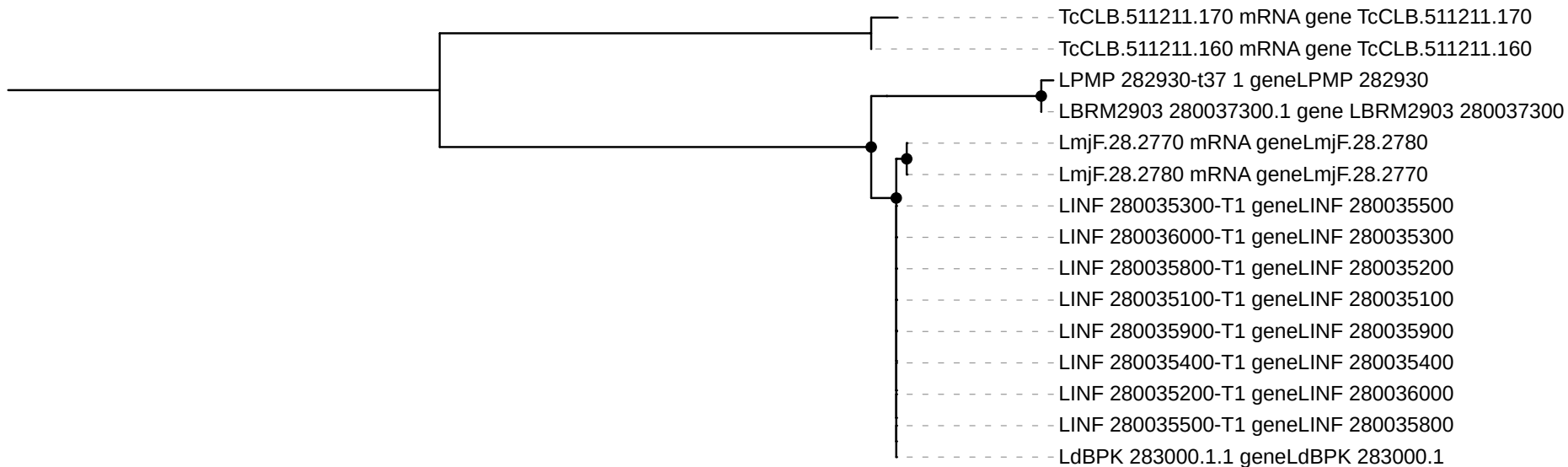

Supplement: Supplementary file 5 — Additional file 5: Figure S3. Phylogenetic relationship between the different HSP70 gene copies in trypanosomatids. The figure represents the phylogenetic analysis of HSP70-Long sequences in trypanosomatids, as recognized by the primers used in the study. The black dots represent well-supported nodes (Bootstrap ≥ 90). [file 13071_2023_6073_MOESM5_ESM.pdf]

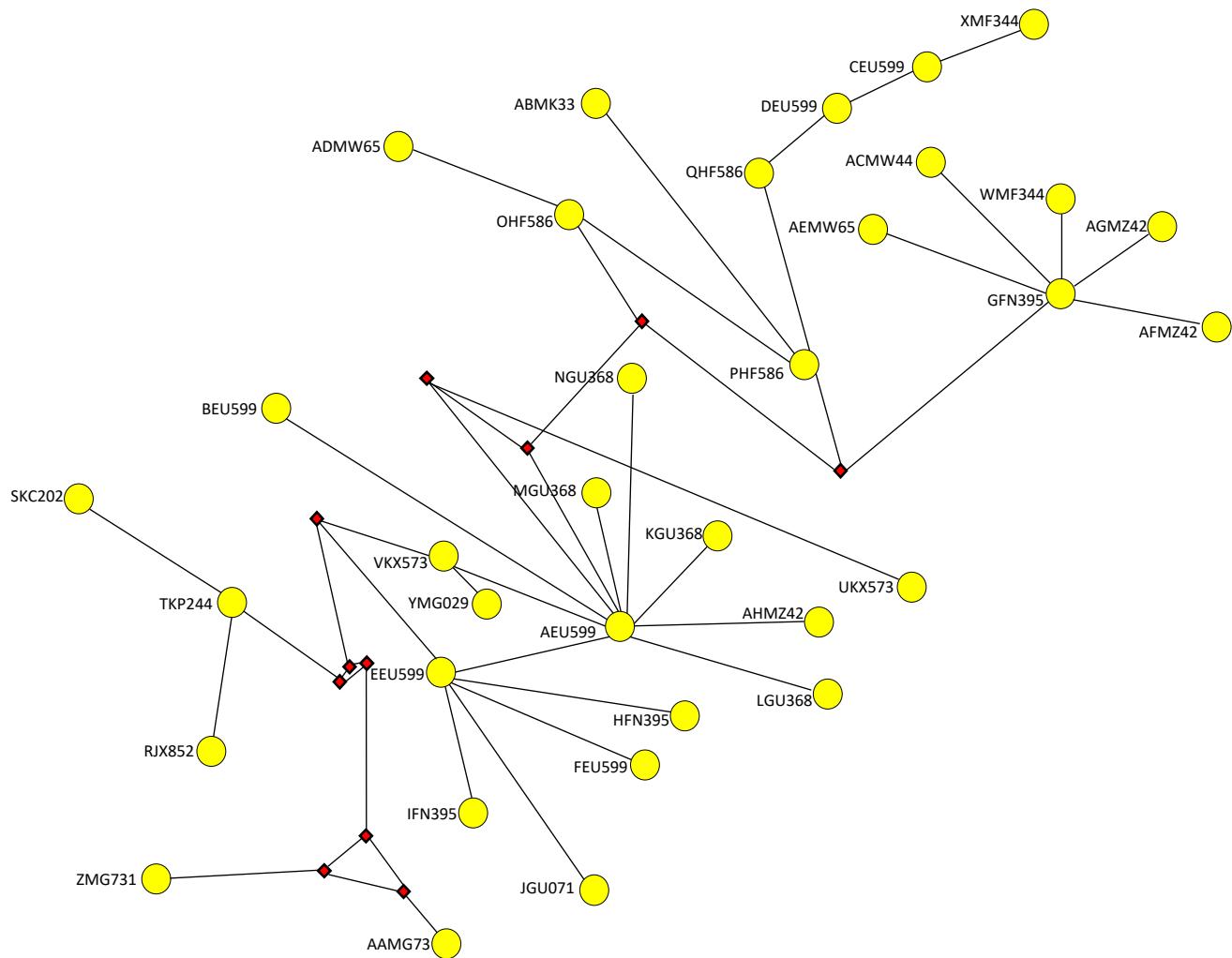

Supplement: Supplementary file 6 — Additional file 6: Figure S4. Network analysis of 34 haplotypes from HSP70-Long reference sequences. Each sequenced haplotype is represented by a circle. Black lines on the branches indicate the mutational changes between the different haplotypes. [file 13071_2023_6073_MOESM6_ESM.pdf]

# A

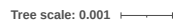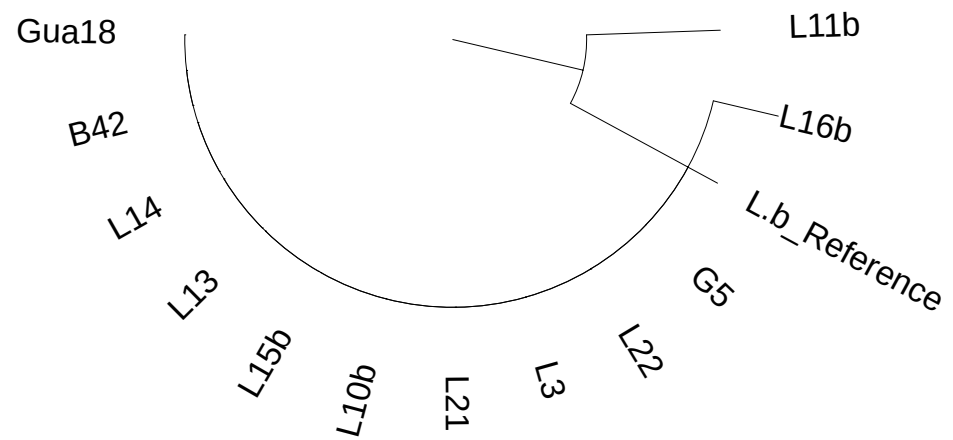

# B

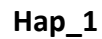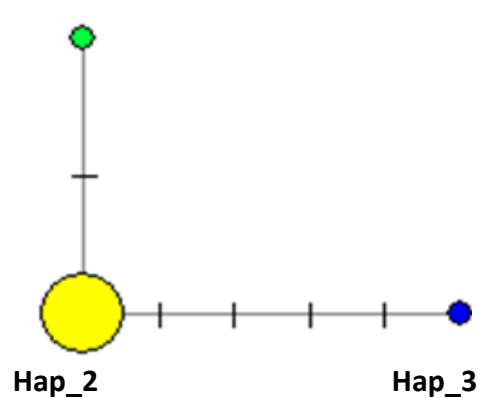

**Hap\_2**

**Hap\_3**

# C

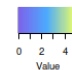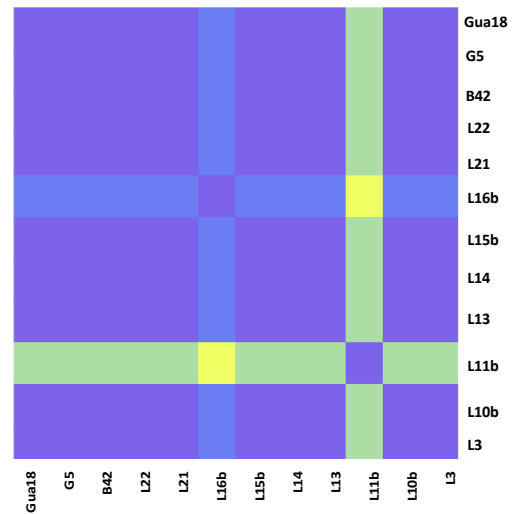

Supplement: Supplementary file 9 — Additional file 9: Figure S5. Analysis of intraspecies diversity among L. braziliensis sequences. A Phylogenetic relationship among L. braziliensis hsp70 sequences. B Haplotype of L. braziliensis network based on hsp70 sequences. Each haplotype is denoted by a circle and mutational steps between haplotypes are depicted by the number of lines. C Heatmap illustrating pairwise comparison of L. braziliensis sequences. [file 13071_2023_6073_MOESM9_ESM.pdf]
