# Supplementary material for: Validation of Oxford nanopore sequencing for improved New World Leishmania species identification via analysis of 70-kDA heat shock protein
Source: Parasit Vectors. 2023 Dec 18;16:458. doi: 10.1186/s13071-023-06073-9 (PMC10726620; doi:10.1186/s13071-023-06073-9)
Supplement: Supplementary file 7 — Additional file 7: Table S3. Number of reads obtained from HSP70-Long- and HSP70-Short amplicon-based MinION™ sequencing for each sample included in the study [file 13071_2023_6073_MOESM7_ESM.docx]

**Table S3.** Number of reads obtained from HSP70-Long and HSP70-Short Amplicon-base MinION sequencing for each sample included in the study.

| **Sample ID** | **Number of reads** | |
| --- | --- | --- |
|  | **HSP70-Short** | **HSP70-Long** |
| BON-L3 | 1,198 | 1,571 |
| BON-L10 | 2,833 | 439 |
| BON-L11 | 947 | 137 |
| BON-L13 | 3,262 | 254 |
| BON-L14 | 2,698 | 4,099 |
| BON-L15 | 5,037 | 2,324 |
| BON-L16 | 1,559 | 177 |
| BON-L21 | 4,345 | 1,745 |
| BON-L22 | 2,588 | 1,826 |
| BON-L42 | 1,581 | 262 |
| GUA-L05 | 1,450 | 153 |
| GUA-L18 | 3,834 | 753 |
| LCL_005 | 678,302 | 172,324 |
| LCL_009 | 654,420 | 204,869 |
| ARB_006 | 630,831 | 84,476 |
| L14_Ven | 77,900 | 134,027 |
| L15_Ven | 91,701 | 111,229 |
| L16_Ven | 96,233 | 4,373 |
| L17_Ven | 100,655 | 104,314 |
| L18A_Ven | 99,489 | 52,179 |
| L18B_Ven | 114,384 | 68,010 |
| L19_Ven | 120,236 | 92,585 |
| L20_Ven | 114,567 | 77,666 |
| L21_Ven | 108,323 | 73,226 |
| L22_Ven | 105,332 | 105,620 |
| L10_Ven | 609,651 | 77,807 |
| Luka | 117,532 | 79,711 |
