# Supplementary material for: Validation of Oxford nanopore sequencing for improved New World Leishmania species identification via analysis of 70-kDA heat shock protein
Source: Parasit Vectors. 2023 Dec 18;16:458. doi: 10.1186/s13071-023-06073-9 (PMC10726620; doi:10.1186/s13071-023-06073-9)
Supplement: Supplementary file 8 — Additional file 8: Table S4. Comparison of the results obtained between the HSP70-Long- and HSP70-Short amplicon-based MinION™ sequencing, in each sample included in the study. [file 13071_2023_6073_MOESM8_ESM.docx]

**Table S4.** Comparison of the results obtained between the HSP70-Long and HSP70-Short amplicon based MinION sequencing, in each sample included in the study.

| **ID Genome** | **Amplicon-base NGS (HSP70-Short)** | **Amplicon-base NGS (HSP70-Long)** |  |
| --- | --- | --- | --- |
|  |  |  |  |
| BON-L3 | *L. braziliensis* | *L. braziliensis* |  |
| BON-L10 | *L.braziliensis/L.panamensis* | *L.braziliensis/L.panamensis* |  |
| BON-L11 | *L.braziliensis/L.panamensis* | *L.braziliensis/L.panamensis* |  |
| BON-L13 | *L. braziliensis* | *L. braziliensis* |  |
| BON-L14 | *L. braziliensis* | *L. braziliensis* |  |
| BON-L15 | *L.braziliensis/L.panamensis* | *L.braziliensis/L.panamensis* |  |
| BON-L16 | *L.braziliensis/L.panamensis* | *L.braziliensis/L.panamensis* |  |
| BON-L21 | *L. braziliensis* | *L. braziliensis* |  |
| BON-L22 | *L. braziliensis* | *L. braziliensis* |  |
| BON-L42 | *L. braziliensis* | *L. braziliensis* |  |
| GUA-L05 | *L. braziliensis* | *L. braziliensis* |  |
| GUA-L18 | *L. braziliensis* | *L. braziliensis* |  |
| LCL_005 | *L. amazonensis* | *L. amazonensis* |  |
| LCL_009 | *L. amazonensis* | *L. amazonensis* |  |
| ARB-006 | *L. amazonensis* | *L. amazonensis* |  |
| L14_Ven | *L. mexicana* | *L. mexicana* |  |
| L15_Ven | *L. mexicana* | *L. mexicana* |  |
| L16_Ven | *L. mexicana* | *L. mexicana* |  |
| L17_Ven | *L. mexicana* | *L. mexicana* |  |
| L18A_Ven | *L. mexicana* | *L. mexicana* |  |
| L18B_Ven | *L. mexicana* | *L. mexicana* |  |
| L19_Ven | *L. mexicana* | *L. mexicana* |  |
| L20_Ven | *L. mexicana* | *L. mexicana* |  |
| L21_Ven | *L. mexicana* | *L. mexicana* |  |
| L22_Ven | *L. mexicana* | *L. mexicana* |  |
| L10_Ven | *L. mexicana* | *L. mexicana* |  |
| Luka | *L. infantum* | *L. infantum* |  |
